# On Fatty Degeneration

**Published:** 1854-09

**Authors:** 


*On Fatty Degeneration.* By J. W. C. Ely, M. D. (The Annual discourse delivered before the Rhode Island Medical Society, Providence, June 29, 1853. Published by request of the Society.)

All animal tissues are liable to transformation, and as the new tissue is usually inferior in chemical composition and in physical and vital properties to the old, this transformation has been very properly called degeneration. Dr. Williams, in his *Principles of Medicines,* specifies four kinds of degeneration, the fibrous, the granular, the fatty, and the osseous. Of these kinds of degeneration, allow me to call you attention to the third, namely, *fatty degeneration.* Much light has been shed on this diseased condition of the animal tissues from the recent labors of Williams, Paget, Rokitansky, Quain, Barlow, and other distinguished microscopic pathologists. The results of these researches are scattered over the pages of the various medical journals. A summary of what is known on this subject, I hope, will not prove uninteresting to the Fellows of this Society.

Fatty degeneration is a trranformation of normal tissue into molecular fat-granules. This change has been observed most frequently in the muscular and fibrous tissues, and in the various secretory organs of the body, but is not confined to them.

Muscle thus affected is of a pale yellowish fawn color, compared by Laennec to dead leaves; sometimes it is of a dirty pink color; when occurring in patches, it gives to the muscle a nottled appearance. The muscle is soft, flabby, and easily torn, and presents on section a granular instead of the normal fibrons appearance.

Rokitansky (in his *Manual of General Pathological Anatomy,*) thus describes the physical appearances of the fatty changes in muscle.

"The earliest change is one of color. The muscle has a pale-redish appearance; and is found, on close examination, to be not uniformly discolored, but stained irregularly of a yellowish or fawn color, as well marked with longitudinal pale-redish streaks which follow the course of the fibres. As the stains of fat increase in size and coalesce, the muscle acquires an almost uniform fawn color; but its fibrous arrangement still remains distinct. With the advance of the disease it becomes altogether of the color of fat, sometimes being yellow, and sometimes remarkably white, and resembling, accordingly, either tallow or spermaceti. No trace of its fibrous structure remains except some of its tendons or the cellular sheaths of its fasciculi. Up to this stage of the disease, the outline of the muscle has been preserved; but, in the last stage, the mass of fat into which the muscle is changed, mixes with the adipose structures around it. We may, then, find in a limb nothing of its muscles but remnants of tendons and aponeuroses, with their prolongations inwards."

In order to get a clear idea of the appearances presented under the microscope, by degenerated muscular fiber from the commencement to the completion of the change, it will be better to enter a litle into the minute anatomy of muscle. Muscles are made up of bundles of primitive fibres, called fasciculi; each fasciculus is encased in a transparent, tough, elastic membrane, or sarcolemma. These fasciculi are surrounded and held together by areolar tissue, which gives support to the capillaries and nerves of the part. The fasciculi present distinct transverse markings, and less distinct longitudinal ones. In some fishes, as the eel, the fasciculi cleave transversely into disks; in other classes of the animal kingdom, as the mammalia, they cleave into fibrillæ.

Under the microscope the disks appear granular, as they are made up of a transverse section of all the fibrillæ. When the fibrillæ are examined with a sufficiently high power, each fibrillæ appears made up of a line of rectangular cells, each cell containing a particle of muscular substance, named myoline.

"Before fatty degeneration commences in voluntary muscle," says Quekett, in his *Lectures on Histology*, "the transverse striæ disappear; and I have long known that the first trace of this disease is marked by a disturbance of the particles of myoline, which appear as so many very minute granules, scattered irregularly within the sarcolemma, leading one to suppose that the delicate cell around each particle had given way, thereby allowing the myoline to escape, and destroying all regularity both of the transverse and longitudinal markings. As the disease progresses, the myoline is replaced by minute highly-refracting globules of oil, until at last the whole sheath is full of them."

Mr. Hallet (*Edinburgh Medical and Surgical Journal*, 1849,) found that a piece of the greater pectoral muscle, weighing one ounce, which had undergone fatty degeneration, yielded seven drachms and one scruple of oily matter. He also states that the outer fibres of a muscle first undergo fatty degeneration; the inner ones becoming affected in time.

Complete fatty degeneration produces no diminution in the size, or change in the form of muscles thus affected.

The following (condensed from the 16th No. of *Ranking's Abstract*) illustrates this form of fatty degeneration:

Mr. Erichson amputated the leg of a woman, aged 48 years, of a leucophlegmatic temperament; for fourteen years she had suffered from abscesses in and about the foot, which resulted in necrosis of the bones of the foot and disease of the ankle-joint. The calf of the leg was not much wasted; upon examination, the muscles appeared of their normal shape and size, put presented a pale fawn color and almost waxy aspect, instead of the healthy redish-brown. This change was most complete in the superficial muscles; a few healthy muscular fibres were found in the deeper layer. The same change was observed in the muscles of the sole of the foot. The microscope proved the change to be far advanced fatty degeneration. The stump healed kindly, thus showing that degenerated muscles will heal as well as healthy ones.

The following is from the January number of the *American Journal*, for 1853:

A man, aged 60, was admitted into the Massachusetts Hospital with an extensive ulcer of the leg. When twenty-four years of age, he cut into the cavity of the knee-joint with a broadaxe. After four months' confinement, he recovered with an anchylosed joint; at thirty, the ulcer commenced and continued open to the time of admission. The leg was amputated by Dr. S. D. Townsend. Upon examination, almost the whole gastrocnemius muscle was found to have undergone complete fatty degeneration.

Mr. Toynbee has found fatty degeneration of the tensor tympani muscle, even in children, in those cases in which the function of the tympanum was imperfectly performed, from inflammation or any other cause.

. The following interesting case of general fatty degeneration of the voluntary muscles was reported to the Royal Medical and Chirurgical Society, Dec. 9, 1851, by Dr. Meryon:

G. H. P., from the time of birth till four years of age, was perfectly healthy; at this time he began to manifest some weakness of the legs, which continued to increase uninfluenced by remedies; at eleven, he could neither walk nor stand; in a similar manner he lost all muscular power in his arms. At seventeen, he was attacked with a simple fever, and died. Previous to examination, the disease was supposed to be in the spinal cord. The limbs appeared remarkably well developed. Upon examination all the viscera of the head, chest, and abdomen were perfectly healthy, but the muscles through the whole body were atrophied, soft, and nearly bloodless, of an ochre color, and flabby. The microscope showed that they all had undergone fatty degeneration. Two younger brothers began to manifest the same symptoms and condition at the same age; the fourth had not arrived at the age when the others were attacked. There were six daughters in the same family, but none had presented any symptoms of the disease. Dr. Meryon mentions another family of two sons and two daughters, in which the sons were similarly affected, and the daughters escaped. Also a third family, of three sons and one daughter, the sons alone affected. Sir Benjamin Brodie mentions three or four like cases, all which occurred in males.

Dr. Meryon regards the following symptoms as diagnostic of this disease: 1. Absence of all symptoms, indicating active nervous disturbance, as pain in the back and head, rigidity of the trunk, or tremulous motions of the limbs, &c. 2. The very slow and almost impreceptible loss of power. 3. The perfect command over the bladder.

The course of treatment he pursued with his surviving patients, and by which they were improved, was: 1. To supply to the blood those fibrinous elements in which it was deficient; 2. Exciting to action the capillaries of the muscular fibres by exercise; 3. Communicating to the muscles artificial electric currents. He found the interrupted current more beneficial than the continuous. (*Lancet*, Dec. 20, 1851, p. 588.)

Mr. Hallet (in the April number of the *Edinburgh Midical and Surgical Journal*, for 1849,) published an account of a similar case of fatty degeneration of nearly all the voluntary, muscles. Of the involuntary, the heart alone was degenerated. The patient, a man, died at the age of seventy-eight.

Fatty degeneration of muscular fibre is not confined to man; it occurs often in the lower animals. If fibres from the breast or wings of fowls are examined under the miscroscope, some of the fasciculi will be found to have undergone this degeneration. This arises, no doubt, from their want of use. Quekett mentions having observed the same change in the muscles of the legs of ostriches, after being long kept in close confinement; the degenerated fasciculi are whiter then the more healthy. He has also observed the same degeneration in the bones of this animal.

Mr. Kent has reported (in the *Veterinary Record*, for 1847,) a case of rupture of the right ventricle of the heart, in a horse, from fatty degeneration.

Adipocire is an instance of fatty transformation of the animal tissues after death. For its production, it is necessary that the tissues be exposed to the action of a certain amount of moisture and kept excluded from the air.

Under the microscope, the areolar is the only tissue seen. Numerous instances of the conversion of the human body into adipocire have been published. In one of the burial-grounds of Paris, fifteen hundred bodies were interred in one pit; most of them, by the action of water, were converted into adipocire. Under favorable circumstances, the same change has been noticed in the vaults connected with dissecting-rooms.

Fatty degeneration must be distinguished from fatty accumulation or substitntion. Fatty accumulation is a hypertrophy of the normal adipose tissue or fat-cells; it arises from an excess of nutrition; degeneration results from deficient nutrition. Fatty accumulation may, by its bulk, press upon and cause the wasting of the surrounding tissues, and thus be substituted, as it were for them. Quekett mentions a mutton chop, preserved in the Museum of the Royal College of Surgeons, in which nearly all the muscular substance is replaced by adipose tissue. He also cites the following case, which occurred a year or two since: The leg of a man, aged thirty-five, which had been palsied since he was three years of age, was amputated. Upon examination, only a few fasciculi of voluntary muscular fibre, and those of very small size, could be found, the mass of muscles being replaced with adipose tissue. The few fibres that were found were perfect healthy, differing in this respect from true fatty degeneration.

Granular degeneraticn might easily, upon a superficial examination, be mistaken for fatty degeneration. According to Williams, a tissue having undergone granular degeneration presents under the microscope a great increase of aggregated granules, with a corresponding diminution of the proper fibrous tissue; It is not so opaque, or easily torn, or soft as true fatty degenerated tissue; the granules are not so highly refractive, and no free oil-globules are mingled with them.

Paget, in his Lectures on Nutrition, Hypertaophy, and Atrophy, describes a form of granular degeneration of the muscular fibres of the heart, which appears to be intermediate between the above and true fatty degeneration. The heart has its normal size and form; to the feel it is soft, doughy, and inclastic, can be easily moulded into any shape; in a word, it has the physical appearance of a heart in which decomposition has commenced; it has not the bright ruddy brown color of a healthy heart, but is of a duller, dirtier, lighter brown, which, in some parts, shades off into a pale fawn color. If the wall of the left ventricle be partly cut through, the rest is easily torn. The cut surface appears granular. The microscope shows that the primitive fibrillæ are inclined to divide transversely, as in certain fish, rather than longitudinally, as in man. This transverse cleavage gives the torn muscle its granular appearance. Minute oil particles are seen within the sarcolemma of the fibre. In three cases, in which Mr. Paget found this diseased condition of the heart, the death was sudden and preceded by symptoms referable to the heart. Persons leading a quiet, inactive life, may have this diseased condition of the heart, without its giving rise to any noticeable symytoms, but they would probably quickly succumb to the shock of an accident, a severe operation, or any violent sickness. Does not this diseased condition of the heart account for many cases of sudden death, where a hasty examination has failed to detect disease sufficient to account for death? But future researches will probably reveal that the different forms of granular degeneration, described by Williams and Paget, are simply different stages in the process of true fatty degeneration, probably the commencing and intermediate stages.

The following case, reported (to the Pathological Society of London,) by W. F. Barlow, illustrates this form of granular degeneration; and, as far as one case goes, it shows that granular degeneration is only an antecedent stage of fatty degeneration:—

J. B., cabman—precise age not known, evidently very old; the arcus senilis existed in both eyes, more strongly marked in the left; of temperate and active habits; with the exception of one day, had attended regularly to his duties; muscular system well developed. The manner and suddenness of his death were not known; when seen, the cab was moving along the street, he lying back upon the seat dead; the reins had fallen from his hand. Examination twenty-four hours after death: Body rigid; skull uncommonly thick; arachnoid opaque; brain shrunken; convolutions small; gray matter thin and pale; the fibrous or white part of brain tough; even septum lucidum and olfactory nerves not easily torn; arteries of the brain full of atheromatous patches; the left vertebral artery contained a cylindrical mass of fibrin a third of an inch long—thus diminishing the amount of blood to the brain—at this point the coats of the artery were badly degenerated.

The heart was flabby; did not preserve its rounded form, but lay flattened; had a dingy color, but not the fawn color and mottled appearance of fatty degeneration; the walls were softened, especially those of the left ventricle; the cavities were dilated, also the aorta, which was thickly studded with atheroma; aortic valves were expanded and thin; the coronary arteries were much degenerated, but their caliber was not diminished. The muscular fibres of the heart were easily broken, and in some spots entirely destroyed; viewed under the microscope, the striæ seemed disappearing in many places; in others, the fibres remained perfect. The degeneration was most extensive in the left ventricle, but it existed in other parts. The degeneration was mostly granular, but the granules were seen here and there to be undergoing conversion into oil-globules.

Dr. Richard Quain, in an article read before the Medico-Chirurgical Society of London, since published in its *Transactions*, and reported in the various medical journals, specifies two forms in which fat occurs as a disease of the heart, namely: Fatty accumulation and fatty degeneration. He gives a table of 15 cases, in which the growth of the normal fat tissue upon and among the fibres of the heart was so great as to become a disease, that is, to impair the structure and functions of the heart, causing derangement of the circulation. Five of the 15 cases had suffered from dizziness or coma; 8 from syncope; 9 from shortness of breath and feeble circulation. In all but one, death occurred sudddenly. One died by coma; 3 by rupture of the heart, and 10 by syncope. This accumulation of normal fat tissue is usually found at the base of the ventricles—along the line that separates them, in the course of the coronary arteries, and on the posterior part of the right ventricle; but occasionally, as in some of the cases reported by Quain, it completely cyvered the organ externally, and penetrated to a considerable depth between the muscular fibres, and the heart looked as if its walls were made of fat. Dr. Hope mentions a case in which a layer of fat, a quarter of an inch thick, covered the anterior and lower half of the right ventricle. Although in such cases the walls are attenuated, pale, and flabby from pressure, yet the fibres upon examination will be found unchanged in structure. The tendency of this form of fatty heart is to end in dilation of one or more of its cavities. Hope gives three cases in which he suspected fat to be the cause of disease; in only one was he able to verify his suspicion by dissection. The other two presented the same symptoms, namely, enfeebled sounds, especially the first; irregular pulse, without valvular disease; and oppression, or even pain in the præcordial region, with signs of retarded circulation producing cerebral, hepatic, and other congestions.

Dr. T. K. Chambers, in his *Gulstonian Lectures*, has collected the histories of 38 obese persons. From these, it appears that the most common age, at which corpulency begins, is from 18 to 30. In 21 of the 38 cases, it began between these ages. In most of the cases there was an hereditary disposition to corpulency. Sex seemed to exercise no influence, as it occurred in 19 males and the same number of females. Dr. Chambers found partial obesity is very apt to occur about the time of the cessation of the functions of the generative organs, being deposited in the omentum and upon the heart more commonly than under the subcutaneous tissue. This excessive overloading of the heart with fat occurs occasionally in those with no tendency to corpulency in other parts; but, of course, is much more frequent in those that have the tendency. Out of 36 corpulent persons who died at the St. George's Hospital between 1845 and 1850, the hearts of 12, upon examination, were found loaded with fat. Of 165 persons, not notably fat, who died at this hospital during the same period, of disease of the heart, in only 4 was there much fat deposited on the heart. The tendency to partial deposition of fat at the turn of life increases with the increase of years.

Dr. Chambers gives the cause of death in 69 obese persons, as shown by *post-mortem* examinations made at the St. George's Hospital: Of 57 whose hearts were examined, 50 were found diseased, namely, 5 hypertrophied; 8 hypertrophied and dilated; 26 dilated; and 11 atrophied. In 13 of those which were dilated, 2 of those atrophied, and 1 of those hypertrophied and dilated, thet is in 16, there was an increased quantity of normal vesicular fat about the base of the heart. Of the 11 cases of atrophy, all that were submitted to the test of the microscope were found to have undergone fatty degeneration.

In the second form of disease of the heart from fat, there is not of necessity any deposit of fat upon the heart or among its fibres; but the muscular tissue is disintegrated into fat granules or moleculs—a true process of decay. By accident, Dr. Quain discovered changes produced in the heart of the dead similar to those that often occur in the living, whose vitality is low, circulation feeble, nervous system deranged, and tissues damaged by atrophy. He and others also have since instituted experiments for the conversion of albuminous and fibrinous textures into adipocire; and from chemical and microscopic observation, both of adipocire and degenerated muscle, have concluded that the appearances in the artificial and natural processes are identical. The observations of Quain, Williams, Paget, and Rokitansky, all go to establish the conclusion, that when the vital properties of the protein compounds are impaired, they succumb to the physical forces around them, and assume the more simple forms common to animals, plants and minerals.

Imperfect nutrition is the first step in the process of fatty degeneration dependent upon original weakness or deficient action of the assimilating organs. In the case of the degenerated heart, it may suffer, in common with other organs of the body, from mal-nutrition; or, locally, from disease of the coronary arteries or the ulterior effects of endo and pericarditis.

The most frequent seat of fatty degeneration of the heart is in the walls of the left ventricle, less often in the septum, sometimes in the right ventricles, and more rarely in the auricle. Out of 68 cases of fatty degeneration of the heart, Dr. Qain found it in connection with hypertrophy in 39 cases. This, he believes, is owing to the less perfect nutrition of hypertrophied hearts. Rokitansky attributes it to disturbance of the balance of the nervous functions. To the same cause, whatever it may be, is due the fatty degeneration of the muscular tissue of hypertrophied bladder, first pointed ont by Mr. Hancock. In the remaining 29 cases, the heart was either of normal size or diminished.

In consequence of diminished consistency of the walls, rupture occurred in 25 of the 68 cases; in 20 the rupture was complete; in 5, incomplete; being limited to the external, internal surface, or in the substance of the heart, the latter constitutes what what has been called cardiac apoplexy. Blood thus effused into the substance of the heart, becoming encysted, and losing its color, simulates an abscess. Cardiac aneurism may result from fatty degeneration of the walls of the heart.

Coma and even apoplexy result from fatty degeneration of the right ventricle; its weakened walls have not the power to propel the blood onward; its cavities fill, and the circulation is obstructed. Faintness and syncope result from the same condition of the left ventricle. It was present in 15 of Dr. Quain's 68 cases. Syncope in these cases may be only transitory, or it may end in death. In 54 of the 68 cases, death was sudden. Out of these 54 sudden deaths, 21 were by syncope.

From our present knowledge of the symptomatology of this disease, the diagnosis must be more or less conjectural. We have no physical sign or symptom pathognomonic of the change, unless the senile arc prove to be such. The following are the usual signs and symptoms: Feeble impulse of the heart, feeble first sound, extended dullness on percussion, perhaps a murmur with the first sound, or imperfection in the second sound of the heart. The pulse is never strong. Of Dr. Quain's 68 cases, in 13 it was irregular; in 14 weak; in 8 slow; and in a few, full, regular, and quick. The countenance is often anxious. Mental irritability usually exists; copious sweats from slight causes; feeling of fatigue from trifling exertion, especially upon going up hill; breathlessness, slight at first, but gradually increasing, till any exertion will induce the most intense suffering. Besides breathlessness; syncope and pain in the region of the heart are frequent symptoms; these three may occur singly or together. Many of these symptoms are the symptoms of angina pectoris, and it is the opinion of both Quain and Ormerod, both of whom have given much attention to this subject, that in fatty degeneration of the heart is found the true explanation of the pathology of angina pectoris in many cases, especially those in which no disease of the structure of the heart was detected; the fatty degeneration of its muscular tissue being overlooked, and the symptoms all attributed to diseased coronary arteries or fat upon the heart. All these symptoms, of course, are more marked when the heart, from some local cause, has become degenerated than when it participates in the degeneration of the whole body, as in old age.

*Treatment.*—Dr. Quain found the results of treatment more successful than at first might have been supposed. Though we may not be able to restore disintegrated fibres, yet we may prevent the degeneration of those remaining, and render them more effective. To do this, we must improve the condition of the blood; make it richer in fibrin, albumen, and corpuscles, a better stimulant to the heart. The condition of the digestive organs should be particularly attended to. The use of iron has been found especially advantageous. Paroxysms of dispnœa and pain are to be relieved by antispasmodics; their recurrence may be prevented by leeches to the region of the heart, followed by counter irritation. Dr. Quain found narcotics injurious. Exertion and over-exercise should be forbidden. Dr. Kenedy thinks a well-directed system of exercise, active rather than passive, a most valuable means of cure in this disease.

Perhaps the two following cases, reported by Dr. Quain, (to the Pathological Society of London,) will give a better idea of fatty degeneration of the heart than any summary of its symptoms:—

Lady H., aged 58, on rising from bed, went to the night-chair to empty the bladder, then laid down, and died immediately. From her 51st to 54th years she had three attacks of apoplexy, followed by slight weakness of right upper and lower extremities. After the first attack, she began to complain of shortness of breath upon exertion. About fourteen months before death, she began to suffer most distressing and alarming attacks of dyspnœa; these came on usually about two o'clock in the morning; the extremities and face became cold and livid, pulse feeble and irregular; as the sense of threatening suffocation became less, a severe pain in the præcordia and across the chest came on. The impulse of the heart was weak, the first sound low and prolonged, second sound clear, no murmur, extent of dullness over heart increased. The arcus senilis existed in both eyes. Upon examination, traces of the three attacks of apoplexy were found in the brain; the arteries at the base of the brain were studded with atheromatous or fatty matter. The lungs were healthy. The anterior branch of the left coronary artery was extremely ossified, the right auricle dilated and thickened, the right ventricle thickened, the greatest thickness of left ventricle thirteen lines; the valves were healthy. The septum near the apex and the wall of the left ventricle towards the anterior surface of the heart presented the pale buff color of fatty degeneration, which passed into, and contrasted with, the healthy flesh color of other parts of the heart. The microscope revealed the characteristic appearance of fatty degeneration, and, mingled with it, a large amount of fibrous tissue. The liver was enlarged, hard, and granular; kidneys were rough and granular, one enlarged and the other atrophied. Ossification of a branch of the coronary artery, with degeneration of that part of the heart it supplied with blood, clearly points to impaired nutrition as the cause. (Condensed from the *Lancet*, Dec. 14, 1850.)

The subject of the following, a lady of rank, who had, through life, suffered much mental anxiety, died at the age of sixty-eight. For ten years previous, she was nervous, irritable, and often passed sleepless nights; she occasionally had a cough with expectoration, frequently complained of heaviness and oppression in the sternum. She was always complaining, yet never seriously ill. About two years before her death, the pulse was noticed to be irregular and uncertain in action. Two months before her death, she complained of numbness of the fingers of the left hand, and of a tingling and uneasy sensation over the surface of the body generally. Within forty hours before death, she had four very severe attacks of pain, which she called "spasms in the stomach;" the first was attended with a rigor and nausea; in the fourth attack, she shrieked loudly, raised herself in bed, or attempted to do so, and died instantly. Upon examination, the pericardium was found filled with blood, a pint in quantity, partly coagulated, which had escaped from a rupture an inch long in the anterior wall of the left ventricle, parallel to and near the septum. In the ruptured part and near the apex of the heart, the muscular fibre had undergone complete fatty degeneration. In other parts, the muscle was perfectly healthy. That branch of the coronary artery which supplied this circumscribed, degenerated part of the heart, was almost closed by osseous degeneration of its coats. There was but little adipose tissue on the heart, although the body generally abounded with it. The liver was greatly enlarged and fatty; the other organs were healthy. (Condensed from *Lancet*, Dec. 27, for 1851.)

The following case was reported to the Pathological Society of London by Dr. Baly :—

Mrs. M. F., aged 52, above medium height, fat, and of a leucophlegmatic temperament, of temperate habits. She had suffered mentally from mere domestic troubles. A little more than three years before her death, she was attacked with paralysis of right arm and leg, with loss of speech ; there was neither loss of sensation of the affected side nor of consciousness. She recovered an imperfect use of the palsied muscles. She did not complain of short breath, palpitation, or faintness ; but a sense of oppression in the chest, and a frequent desire to draw a longer breath than she could do. Pulse feeble; both impulse and sounds of the heart were feeble. Six days before her death, she complained of pain behind the middle of sternum, which confined her to the bed for three days. The day before her death, she appeared unusually well; the following morning expressed herself as feeling well; while washing herself, she raised her hands and expired instantly.

Upon examination, the basilar artery and the branches forming the sides of the circle of Willis were found opaque, rigid and contracted. The parts of the brain supplied by these arteries alone presented the appearance of disease. The under surface of the crura cerebri was softened to a pulpy consistence for a line in depth; the left half of the pons varolii was flattened and contracted, and showed a loss of medullary substance, which extended to the medulla oblongata. The pericardium was distended with many ounces of serum, and a dark coagulum, which completely enveloped the heart. The base of the heart and right ventricle was loaded with fat for a space three inches square. The rounded part of the left ventricle was of a mottled dull yellow, or drab and pink color, with no appearance of fibrous tissue. In the middle of this part, just behind the left convex border of the ventricle, there was a rupture of an inch, commencing half way from the base to the apex, running towards the apex; nearly on a line, and below this rent were two much smaller ones. The wall of the left ventricle was of normal thickness, its cavity enlarged, and more nearly globular than usual. It was in the dilated part that the rupture occurred. The mitral valve was thickened and opaque, the aortic were also thickened, and their free action hindered by a bony ring at their attached border. In the coronary arteries were numerons opaque and thickened patches of cartilaginous or fibro-cartilaginous structure. A branch of the left coronary, which ran to the degenerated part, was for three-fourths of an inch much enlarged, thickened, yellow, and rigid, and filled with a firm, dark coagulum, and its canal seemed obliterated at the very margin of the degenerated part. The other parts of the heart were healthy. The arch of the aorta presented many patches of atheromatous degeneration.

Various parts of the heart were subjected to microscopic examination; in the septum, which appeared to the naked eye most healthy, a fibrilla here and there were seen degenerated; in portions from the right ventricle, which had a soft and unctuous feel, more fibrillæ were found degenerated; and in parts from the side of the rent scarcely a trace of normal muscular tissue could be seen; nearly all the primitive fasciculi had undergone fatty degeneration. The arcus senilis did not exist. (Condensed from *Lancet*, Jan. 31, 1852.)

This case is particularly interesting and instructive. It cannot be doubted, that the disease of the brain and of the heart was dependent upon the disease and obstruction of the arteries, impairing the nutrition of the parts they supplied. In fact, all the changes in the body may be referred to the diseased condition of the bloodvessels.

The yellow, obaque arc, or zone, called *arcus senilis*, often seen in the eyes of the aged, is another instance of fatty degeneration. It is particularly interesting in this connection, as it is supposed, by some, to furnish a diagnostic mark of the same change in the heart. To Mr. Edwin Canton is due the credit of elucidating the pathology of the arcus senilis. (The results of his researches are contained in the *Lancet* for May 11, 1850, and for Jan. 11 and 18, 1851.)

The senile arc is produced by a gradual transformation or degeneration of the tissue of the membrana, propria of the cornea into fat-globules. From extended observation, Mr. Canton concludes that the senile arc rarely exists in persons under forty years of age. He mentions that he has seen only four exceptions to this rule; one was a sickly girl of sixteen, who had suffered from two attacks of choroiditis in the left eye; in the same eye there existed an arc. According to Mr. Canton's observation the arc rarely exists in one eye alone; where it does, it is owing to some local impairment of nutrition. Fatty degeneration of the ophthalmic artery frequently coexists with the senile arc. Mr. Canton found the upper arc the first to make its appearance. In regard to the senile zone as an index to a similar change in the heart, he makes the following assertion: "I have in no instance found the senile arc, when well developed, unaccompanied by fatty degeneration of the heart. The extent of degeneracy in this organ has appeared to me to bear a relation to the degree to which the cornea was invaded by the deposit." His observations, with the following exception, were confined to those advanced in years, and for a long time inmates of a workhouse: A man, aged 42. died of lumbo-pelvic abscesses; a well-developed senile zone existed in both eyes; *post-mortem* examination proved the coexistence of extensive fatty degeneration of the heart.

Dr. R. Quain mentions the coexistence of fatty degeneration of the heart and of the arcus senilis, in a man only twenty-eight years of age. Dr. C. J. B. Williams gives a table of twenty-five cases; in all, there existed the signs and symptoms of fatty degeneration of the heart; all but three were males, of ages varying from 45 to 77. In all but two the senile arc was to be seen more or less distinctly marked. In two cases, he proved the coexistence of these diseases by *post-mortem* examinations. Dr. Quain has the following more cautious remarks upon this subject: "When the signs and symptoms of fatty degeneration of the heart are present the appearance of the cornea will greatly aid in the diagnosis. It must not, however, be forgotten that fatty degeneration of the heart may occur under circumstances, and at an age when we would not expect to find this lesion of the cornea; so likewise, but in a much less degree, we may expect to find the change of the cornea independently of the change in the heart."

The value of the senile arc as a sign of fatty degeneration in other parts of the body depends upon its symmetrical development in both eyes. When it exists in one alone, it is due to some disease or accident which has impaired the nutrition of that eye alone.

The observations of Dr. E. B. Haskins, of Clarksville, Tenn.* are opposed to the conclusions of Mr. Canton. The number of cases is twelve, being all that came under his notice during three months. In his opinion, only two had any symptoms referable to the heart, and in one of these the palpitation was evidently hysterical. One had consumption, one dyspepsia, one paralysis agitans, and one other neuralgia; the other six were healthy. Three were under forty years of age, one was only twenty-seven and of remarkable health and vigor, the oldest was ninety. Dr. Haskins found that the lower arc began first and was most fully developed.

Mr. Dalrymple states that the microscope reveals many oil-globules, and sometimes plates of cholesterin in the milky liquid of *soft cataract.*

One of the most interesting facts connected with this subject is that brought to light by the researches of Mr. Cilian, and confirmed by the observations of Mr. Rainey, it is this that the normal process of restoration of the uterus, after parturition, to its ordinary state, is effected by the conversion of the now useless muscular tissue into oil or fat granules.

Mr. Rainey has given (in the January number of the *Lancet,* for 1853,) the results of the microscopic examination of the uteri of two women; one died three weeks after delivery; the other, aged thirty-six, died four weeks after delivery, of metritis and purulent absoption. Thin sections of the uterus, in both, exhibited unequivocal marks of fatty transformation of the muscular fibres; but it was better marked in the last, as might be expected, as a longer time had elapsed from the confinement of the patient to her death. The opinion of Mr. Rainey is, that this "change is most probably a normal one, and that it occurs in the muscular fibres of the uterus in all cases after delivery."

Fatty degeneration of the placenta should be mentioned in this connection. Dr. Robert Barnes read before the Medico-Chirurgical Society of London, May 13, 1851, a paper on this diseased condition of the Placenta; and its influence, as a Cause of Abortion, death of the Fœtus, Hemorrhage, and Premature Labor.

In this paper, Dr. Barnes gives two cases of premature delivery, both at seven months; in the first there was hemorrhage at the third month, and at the time of delivery; in the second there was no hemorrhage. In both, the fœtus had been dead some time before labor commenced. Dr. Barnes describes the appearance of the placentæ thus: "The uterine surfaces were studded with fatty masses, varying in size from that of a bean to one mass,

*American Journ. Med. Sci., Jan., 1853.

†Lancet, May 26, 1851.

which was as large as a pigeon's egg." These masses were firm, of a yellowish white, and bloodless.†

Dr. Hassall subjected to microscopic examination thin sections of these masses, also parts of the apparently healthy placenta; in the former, the degeneration was found complete; in the latter it had commenced. The oil granules were found in the chorion, the walls of the unbilical capillaries, and in the spaces between them. No blood was found in the vessels. Dr. Barnes thinks fatty degeneration of the placenta causes death of the fœtus by cutting off its necessary supply of blood. Dr. Barnes claims that, with the exception of a similar case recorded by Professor Kilian, his are the only recognized cases of fatty degeneration of the placenta reported.

Dr. Barnes read before the same Society, Feb. 22, 1853, a farther account of this degeneration. This paper contains the history of several cases observed since the publication of the former one. In the first case, the fœtus and placenta were expelled together, between the fifth and and sixth months of pregnancy. Though, probably, some time dead, yet there was no sign of decomposition in fœtus or placenta. The uterine surface of the placenta resembled the outer surface of the brain, both in color and its divisions. There was no trace of healthy placenta. The lobes were of a pale yellow and glistening appearance, the sulci of a pinkish red, and along these lines the placenta drew an imperfect nutrition from the uterus. The degeneration had been gradual; and so completely had it arrested the circulation, that there was no hemorrhage at the time of delivery. The microscope showed that the vilii were much changed, the chorion mostly destroyed, and the nuclei in the coats of the capillaries enlarged and loaded with fat-granules.

The four other cases reported by Dr. Barnes, of premature delivery, the placenta presented the same appearance of fatty degeneration. Delivery took place from the fifth to the eighth month; in each the fœtus had been some time dead. In two other cases, the abortion happened in the early months of pregnancy; was attended with some hemorrhage; degeneration of the placenta had not proceeded as far as in the others. In the last case, the child was born at the full time, alive and healthy. Fatty degeneration affected the placenta partially; but this change, probably, commenced near the close of pregnancy, and did not so interfere with the nutrition of the child as toprevent its reaching the full time. Dr. Barnes states, from his observations, that when fatty degeneration of the placenta is met with at the full time, it is usually found in the margin of the placenta, small in amount; not sufficient to change the structure of the tissue, or impede its functions. Dr. Barnes found the villi seldom affected by calcareous degeneration; and except in very extreme cases, it did not interfere with the functions of the placenta.*

Dr. Robert Druitt read before the same society, Jan. 25, 1853, a paper on Degeneration of the Placenta at the end of Pregnancy. The conclusions of Dr. Druitt are drawn from the microscopic examination of thirty placentæ taken from women whom he had consecutively delivered. In all, he found either earthy or fatty degeneration to a greater or less extent. He doubts whether a placenta at the full time can be found, free from traces of fatty and earthy degeneration; they may not, and usually are not found together in the same

*Lancet, March 5, 1853.

part, and to an equal amount. As the result of his investigations, he draws the following conclusions. 1. That incipient degeneration was a normal condition of the placenta at the end of pregnancy. 2. That it arose from partial cessation of the active functions of the organ, when the fœtal development was nearly completed. 2. That when it occurred in the earlier months, it probably arose from some antecedent want of nutritive force in the fœtus, or by its death.*

The analogy, that organs which have arrived at the fullness of their growth and the fulfillment of their offices began to degenerate and give signs of incipient death, like the falling of the leaf in autumn, is hardly applicable to the placenta; for as the fœtus increases up to the time of delivery, it of necessity requires an increasing placenta, with the exercise of its full and perfect functions. Numerous and accurate microscopic observations, by capable observers, alone can harmonize the apparently contradictory results of the conclusions of Drs. Barnes and Druitt.

From facts at present in our possession, it is propable that a certain amount of fatty degeneration of the placenta, especially in its margin, can exist near the full term without interfering with its healthful and complete functions. In cases of premature labor, with fatty degeneration of the placenta, in some this fatty change of the placenta is probably the primary trouble and cause of death of the fœtus, and of abortion; in others, this degeneration is the result of atrophy, or death of the ovum.

*Fatty Degeneration of the Arteries.*—Mr. Gulliver first demonstrated that the atheromatous patches, so often seen in the arteries, especially in the larger ones, consist of fatty matter, formed partly by degeneration of the middle or elastic coat, and partly deposited in granules and oil-globules, with scales of cholesterin, under the lining membrane. The elasticity of the artery being thus destroyed, and its strength impaired, dilation, aneurism, and rupture readily follow.

The fact that apoplexy is, in many cases, the effect of this atheromatous degeneration of the larger cerebral bloodvessels, and not of the congestion and escape of blood from healthy vessels, has been often noticed. Many cases illustrating this connection have been reported. The following case occurred at the Dexter Asylum in this city:—

Wm. B. M., aged sixty, married, of intemperate habits, by occupation a shoemaker, admitted into the asylum August 27, 1851. As he was delirious at the time of his admission, and continued so until his death, no history of the attack, or of his health previous to it, could be obtained. There was perfect paralysis of the left side; head hot; face deeply suffused; pupils natural; extremities inclined to be cold; tongue loaded with a yellowish-white fur; pulse seventy, full and hard. On the 29th, the palsied leg and arm were œdematous; tongue dry and brown; pulse small; delirium violent, with extreme restlessness. He died August 30. Upon examination, the coats of the arteries at the base of the brain were found studded with atheromatous patches. The arteries most diseased were the anterior cerebral and communicating. In the anterior lobe of the right hemisphere, near the base, a dark clot was found, and the brain-substance around it much softened; the arteries supplying this part were those most diseased.

---

*Lancet, Feb. 12, 1853.

Mr. Paget has recently pointed out this important connection of apoplectic seizures with fatty degeneration of the coats of the smaller bloodvessels of the brain. (The eleventh number of *Ranking's Abstract* contains a report of his communication to the Abernethean Society, in which are given the microscopic appearances in the cases illustrating this connection.)

According to Mr. Paget, in the earliest stage of this degeneration, "minute shining, black-edged particles, like molecules of oil, are thinly and irregularly scattered beneath the outer surface" of the capillaries, and of arteries and veins of small size. As the disease advances, the whole surface of the affected vessels becomes thick set with oil particles. The size of these particles also increases; in some vessels the granules are conglomerated into clusters of various shapes and sizes, instead of being uniformly scattered over their surface. Occasionally, the different branches of the same vessel will exhibit all these forms and degrees of change—the thinly and thickly scattered oil molecules, the clustered patches, and "the large particles like drops of oil."

The structure, and not unfrequently the shape of the vessel, undergo a change as the degeneration becomes more developed. The proper fibrous tissue of the vessels wastes, and finally totally disappears. The vessels "appear like tubes of homogeneous pellucid membrane, thick set with fatty particles." Clusters of fat-granules raise the outer coat of the vessel, and give it an uneven and knotted appearance. Sometimes the oil particles are so numerous as to seperate the cellular and middle coats for distance; and occasionally the smaller vessels are dilated into partial pouches.

The smaller arteries and veins are most liable to fatty degeneration; the capillaries are much less frequently affected.

The muscular or transverse fibrous coat is the first and principal seat of this degeneration in arteries; in veins, it is the corresponding layer; in vessels made up of one single membrane, the fatty particles are deposited in its substance. But, in the advanced stage of the disease, all the coats become involved; their proper tissue wastes, and unites into a single membrane, which is filled with the deposit.

Besides the cases of Mr. Paget, Mr. Barlow,* Mr. Robert Dunn,† and Dr. Babington,‡ have each reported a case of softening of the brain. In all, the microscope revealed far advanced fatty degeneration of the minute cerebral bloodvessels in and near the seat of disease.

The following is a brief abstract of the symptoms and morbid appearances in Dr. Babington's case:—

James N., aged 45, of regular and temperate habits, dark complexion, short and square-built man, general appearance strong and healthy, by occupation a warehouseman; his general health had always been good, had not been subject to headaches, but had frequent attacks of epistaxis, especially after any violent excitement. All his family are healthy; none, to his knowledge, ever suffered from paralysis. He had lately married a young wife.

A day or two before the attack of hemiplegia, he had pain in the back part of his head. On the day of the attack, he had a severe diarrhœa, which was relieved by two or three doses of brandy and opium. In the evening, while taking tea, he was suddenly seized with complete paralysis of motion

* Lancet, May 24, 1851, p. 577.

† Ibid, Oct. 26, 1850, p. 473, and Nov. 2, 1850, p. 499.

‡ Ibid, Oct. 30, 1852, p. 400.

of the left side, loss of speech and increase of pain in head, without loss of sensation or of consciousness. In a few hours he partially recovered from this loss of motion; but on the following morning the paralysis was again complete.

Delirium came on, and he complained of severe pain in the head, also of pain in the palsied side. Upon admission to the hospital, these symptoms continued. The pupils, sight, and muscles of eyes and eyelids were unaffected; sleep natural; appetite good; pulse 72, small, hard, and laboring; mouth drawn towards the right side; left hand and arm little œdematous.

*Treatment.*—Cupping, mercury, and cathartics. On the 34th day from the attack bronchitis supervened. On the 67th day he died. He was delirious through the whole course of the disease.

Upon examination, the convolutions of the brain were found atrophied; the interspaces filled with clear serum; brain firm; ventricles large and dilated, containing but little fluid; the posterior part of the corpus striatum was found softened, flocculent, and of a yellowish-brown color; the anterior portion of the thalamus opticus was also slightly affected. The coats of the large and small bloodvessels contained extensive atheromatous deposits; and this was particularly the case with the arteries supplying the softened portions. The right ventricle was loaded with fat; the left ventricle of the heart was hypertrophied, pale in color, mottled on its inner surface, and its walls had undergone fatty degeneration. The arch of the aorta was dilated and atheromatous throughout its whole extent. The arteries of the body were generally diseased. The kidneys were atrophied, the liver atrophied, and part of it had suffered fatty degeneration.

Dr. Dittrich has observed fatty degeneration of the arteries of the lungs, in cases of pulmonary apoplexy, similar to that described by Mr. Paget, in the arteries of the brain. Dr. Dittrich found, in a large majority of cases of pulmonary apoplexy, this fatty transformation of the coats of the large and small vessels, but more especially of the smallest vessels.

Dr. Charles Shearman has observed the same fatty transformation in the substance of a minute vessel of a degenerated heart.

*Fatty Degeneration of the kidney.*—Dr. George Johnson claims to have discovered the true pathological change in Bright's disease of the kidney. According to his observations, this change consists in a gradual degeneration of the kidney, beginning with an abnormal deposit of fatty-granules in the epithelial, or secretory cells lining the uriniferous tubes, and in the tubes themselves.

Drs. Johnson and Frierichs believe that a small quantity of fat normally exists in the epithelial cells. Dr. Gairdner never has observed it, except the kidney be diseased.

Dr. Johnson explains the atrophy of the kidney, and the presence of albumen in the urine by the mechanical effects of the fatty deposit. As this increases, the circulation through the plexus of veins, that surround the uriniferous tubes, is impeded, and of necessity the capillaries of the Malpighian corpuscles become congested with blood. This congestion of the capillaries is relieved by the serum transuding through their walls, and mingling with the urine, or by rupture of the capillaries, allowing the fibrin and red corpuscles also to mix with the urine. This view makes the congestive stage secondary to that of deposit. Dr. Johnson thinks that upon the greater or less rapidity of the deposition of fat depends the variety of the disease. He also believes the disease to be of constitutional origin, like fatty liver, and tc require constitutional rather than local treatment. Dr. Johnson usually found fatty degeneration of the kidney accompanied by the same change in other organs, especially the liver and coats of the arteries. This abnormal deposition of fat-granules in the Malpighian corpuscles, in the uriniferous tubules and in the interstitial substance, between the bloodvessels and secretory apparatus in Bright's disease, had been observed and described by several German and French pathologists, previous to the publication of Dr. Johnson's paper in the *Medico-Chirurgical Transactions* in 1846.

Mr. Toynbee agrees with Dr. Johnson as to the deposition of fat-globules in the secretory cells and in the tubuli uriniferi; but differs from him in regarding congestion as a necessary antecedent to this change.

Gluge, of Brussels, from observations make in 1840, concludes that the phenomena of Bright's disease are produced by different pathological changes in the kidney; the most frequent changes are fatty degeneration and lesions resulting from inflammation. His *Pathological Histology*, recently translated by Leidy, contains a table of 12 cases of disease of the kidney, in most of which albuminous urine was a symptom; in 7 of these cases, the microscope revealed deposition of fat-granules in the tubuli uriniferi and in the interstitial substance of the kidney. In some of the cases, the tubuli were filled with oil-globules; and in one, the Malpighian corpuscles were distended with molecules. Fatty degeneration of the liver coexisted in several cases.

Dr. Williams, in his *Principles of Medicine*, claims to have described this increase of fat-granules in the kidney before Dr. Johnson even made his researches; but says: "I have never seen in the kidney anything at all approaching to the condition of the fat liver, in which both cells and interstitial textures are completely glutted with oil-globules." He found fatty degeneration of the liver, spleen, and other parts of the body coexisting with fatty kidney.

Simon, in his *Lectures on General Pathology*, thinks the opinion that the accumulation of fat-granules in the tubuli uriniferi, as a primary deposit, and as the proximate cause of the scrofulous form of Bright's disease not supported by conclusive evidence. He thinks the deposit of granules is not sufficient to account for the destruction of the tubules and the serous or fibrinous infiltration of the kidney, which he states, always exists when it is extensively affected with this disease. The tubules of the kidney of the cat, he states, almost invariably, and he thinks, abnormally contain a large quantity of oil; and though the accumulation is much greater than is ever observed in the human kidney, yet it does not often, if ever, destroy the tubules, or interfere with the function of the organ or health of the animal.

Dr. Frierichs (*Ranking's Abstract*, No. 15,) divides the morbid appearances presented by the kidney in Bright's disease into three states: 1. State of hyperæmia. 2. State of exudation and commencing transformation of the exudative matter. 3. State of degeneration and atrophy.

In the first stage or state, the epithelial or secretory cells are not materially affected; but the tubuli uriniferi, especially of the cortical portion, are usually filled with coagulated fibrin. Sometimes, these coagula are simply casts of the tubuli; at other times, epithelial cells, and changed blood-corpuscles are imbedded in them. This state is often attended with effusion of blood from the Malpighian corpuscles or capillary plexus surrounding the tubules. This condition was observed only in violent and rapidly fatal cases. Frierichs gives 20 cases out of 292 deaths from this disease.

In the second stage, the congested condition is less marked, and that of exudation is increased. The fibrinous coagula in the tubuli degenerate into fatty particles. In the Malpighian corpuscles, fibrinous exudation is seen, which also in time degenerates into fat-granules. The epithelium of the tubuls, particularly of the cortical portion, undergoes a complete transformation; its cells lose their proper form, and become more or less infiltrated with fat-granules; thus, it at last loses its characteristic appearance and function, and becomes degenerated into granular detritus and fat: this condition, Frierichs observed in 139 out of 292 cases examined.

In the third stage, the kidney becomes atrophied. The degeneration of the coagula in the tubuli and in the Malpighian corpuscles, and the transformation of the epithelium, are completed; their removal causes collapse of the walls of these structures, and atrophy. In a few instances, atrophy was produced by the contraction of plastic matter effused into the interstitial tissue.

Dr. Frierichs mentions apoplexy, suppuration, cystic formation, calculous deposits, and tubercle, as accidental anatomical changes of the kidney in this disease. He states that in a hundred parts of healthy dried kidney, the fat varies from four and four-tenths to five and five-hundredths. In Bright's disease, it varies from four and four-tenths to thirteen and nine-tenths. Generally, the amount of fat was greatest in the third stage. Chemical analysis reveals less fat than the microscope would lead us to expect; therefore, Dr. Frierichs concludes, we are not justified in naming as fat all those particles which look like it. Dr. Frierichs found the percentage of fat in the kidney of the cat and dog to vary from twenty-seven and two tenths to thirty-two and five-tenths. The animals were in perfect health, and their urine did not contain any albumen. This last view of the pathology of Bright's disease more satisfactorily explains all the phenomena it presents.

*Fatty Degeneration of the Liver.*—A liver that has undergone this change is enlarged, paler, softer, and more unctuous in its feel than natural; of low specific gravity, (sometimes lighter than water,) and greases paper when heated upon it. The hepatic cells, which make up most of the parenchyma of the organ, naturally contain oil-globules. Dr. C. Hanfield Jones, in a very elaborate paper on the *Structure, Functions, and Diseases of the Liver*, (read before the Medico-Chirurgical Society of London, and reported in the August No. of the *Lancet* for 1852,) states, that he found the quantity of oil in the cells to vary greatly in different classes of animals, and also in the same individual. He believes the hepatic cells contain oil, sugar, and a yellow pigment, but no bile; he found bile only in the ducts; that is, the cells contain the elements of bile, but the combination of these elements, so as to make bile, is affected by the ultimate ends of the bile-ducts.

From microscopic examinations in 1841, Mr. Bowman came to the conclusion that fatty degeneration of the liver depends on a simple increase (but sometimes to a very great extent) of the oil-globules normally existing in the hepatic cells. This theory is taught in the works of Drs. Budd and Williams, and Mr. Simon. But Dr. C. H. Jones holds the opinion that it is not a simple increase of the oil-globules naturally existing in the hepatic cells. He found, upon examining the livers of animals fed on oil, that both the cells and intercellular substance were equally gorged with oil-molecules. But in fatty degeneration of the human liver the oil-globules appeared inclosed in an "indistinct and granular, or semifibrous substance;" but not contained in distinct cells. The liver of an animal fed on oily food to produce fatty liver, contained sugar in the cells; but in true fatty degeneration sugar is not found. Also, in true degeneration of the liver the disease affects only the marginal cells of the lobules, so that each lobule is surrounded by a zone of obaque matter; and these marginal cells are not merely loaded with fat particles, but destroyed. No satisfactory reason is known why the degeneration should be confined to the marginal cells. Dr. Budd states that the quantity of oil in this disease sometimes equals the weight of all the other elements of the liver. Though most frequently met with in cases of consumption, yet it occurs in other diseases. Dr. Budd mentions meeting with it in two cases of chronic dysentery, also in one of cancer, and cites another case from Cruveilhier. Rokitansky thinks fatty liver belongs to the tubercular diathesis. He has found this condition of the liver in tuberculous disease of the mesentery, lymphatic glands, bone, and of other organs, even when there were no tubercles in the lungs as well as in consumption. Louis, Budd, and Williams, all agree iu its being most frequently met with in females. Louis found the proportion as high as four to one. Fatty degeneration commonly affects the whole liver equally, yet it sometimes effects a very small portion. This, of course, is dependent on some local cause. Dr. Budd met with three cases in one year.

The *gall-bladder* is another organ, in which fatty degeneration sometimes occurs. When thus diseased, its coats present a thickened and obaque appearance, and under the microscope numerous oil-globules and scales of cholesterin are seen. Phosphate of lime is often found with the fat particles, and, sometimes, to such an extent as to form of the bladder almost a bony cyst. In all these respects fatty degeneration of the gall-bladder is analogous to atheromatous disease of the arteries. This degeneration of the bladder is sometimes complete, at others, only partial. Dr. Budd found it only in the aged, and more frequently in females than in males.

The *spleen* is occasionally found to have undergone fatty degeneration.

Rokitansky states that "the pancreas is liable to excessive accumulation of fat which may terminate in the conversion of the entire organ into one mass of fat. When the disease has attained its extreme limit, a mere pultaceous strip of fat, retaining the general outlines of the gland, is found in its place; only scattered remains of the acini are discoverable; and in the delicate and thinned duct there is a whey-like, fatty fluid. The disease occurs frequently in drunkards, associated with fatty liver."

Gluge, in his *Pathological Histology*, describes and figures two cases of partial fatty degeneration of the *pancreas*. He also describes and represents the same change in the tubuli seminiferi of the *testicle* of a dog, whose other organs were healthy.

Dr. Walshe states that the *human testis* is liable to fatty degeneration. Mr. Curling gives a case of atrophied testicle which had undergone this change.

Dr. C. Hanfield Jones (*British and Foreign Medico-Chirurgical Review*, April number, 1853,) describes fatty degeneration of the cortical portion of the *suprarenal capsules* as of very frequent occurrence in man, and occasionally met with in animals.

According to the observations of Dr. Jones, the normal atrophy of the thymus gland takes place by accumulation of fat in the interstices of the thymus tissue, destroying it by pressure, as in the disease of the pancreas described by Rokitansky. But Mr. Simon describes cases of true fatty degeneration of this gland. Dr. Jones gives one instance of fatty degeneration of the *thyroid gland.*

*Mollities ossium* is, according to the observations of both Paget and Quekett, an instance of fatty degeneration in the osseous tissue. Quekett, from numerous microscopic examinations of bones thus affected, concludes that the diseased process commences in the lacunæ or bone cells, which increase in size by the absorption of the earthy granules from the walls of the cells, the canaliculi disappear, and several cells unite and form a cavity, in which oil-globules are soon deposited. Mr. Paget thinks the fatty matter results "from the conversion of the cartilaginous bases into fat." The walls of bones thus diseased are very brittle, thin, transparent, and so soft as to be easily cut with a knife. Not only the medullary cavity, but all that remains of the cancellated structure of the bone is filled with oil. Mr. Hunter describes bone thus affected, as "resembling a species of fatty tumour, and giving the appearance of spongy bone, deprived of its earth, and soaked in soft fat."

The oil presents various shades of color, being "bright yellow, pink, and deep crimson."

The quantity of fatty matter found in healthy bone is from two to three per cent. A specimen of bone affected with mollities ossium, analyzed by Dr. Garrod, yielded twenty and one-third per cent. of oily matter. Analysis of Lehmann yielded from twenty-nine to thirty-four per cent.

It would appear that two distinct diseases have been described under the name of mollities ossium; true fatty degeneration, and simple softening of bones from absorption of the earthy particles, leaving the true cartilaginous basis, which is soft, tough, and flexible, not brittle, as that described above. Cases of simple softening have been described by Rokitansky, Bruce, Jones, and others. They occur most frequently after childbirth.

Mr. Adams, in a communication to the Pathological Society of London, (Report for 1848-49,) described fatty degeneration of *cartilage.* According to his observations, the solid contents of the cartilage cells are first converted into very minute spherules of oil; subsequently, these spherules of oil coalesce, and form globules of different sizes, which distend the cells; still later, the cell-membrane disappears; and lastly the intercellular matrix undergoes the same fatty degeneration as the cells. The disease commences on the free surface of articular cartilages.

*Causes of Fatty Degeneration.*—For the following reasons, Mr. Paget concludes that fatty degeneration is a form or manifestation of atrophy, the consummation and result of atrophy: 1. The frequent coincidence of fatty degeneration with emaciation and diminution of the size of the part. 2. The existence of fatty degeneration under circumstances which, in other instances, give rise to simple wasting of the same part. 3. The frequent occurrence of fatty degeneration with senile atrophy. 4. The absence of the nucleus (which all physiologists agree is the active agent in the changes which the cell effects) in both atrophy and fatty degeneration. Mr. Paget does not regard the fatty matter, in such cases, as "a new deposit, but as one of the products of the spontaneous transformation of the tissues at the end of their proper periods of vigorous existence; so that this condition represents the state of a tissue remaining unrepaired, after it has fallen into the ordinary course of degeneration." The fat-granules, in degenerated muscular fibre, are frequently seen arranged in longitudinal or transverse lines, like the healthy elements of the fibrillæ. Under the term atrophy, are comprehended all degrees of impairment of nutrition, from the slightest to the most striking wasting of the tissues, seen in consumption and other chronic diseases. True atrophy occasionally coexists with seeming hypertrophy, as in the hypertrophied heart and bladder, already mentioned, where the microscope revealed fatty degeneration of the fibres. Fatty degeneration being the result of impaired nutrition, its causes will be best learned, as Mr. Barlow has suggested, by considering the conditions admitted to be essential to perfect nutrition, and then looking to the results of the failure of these conditions. Mr. Paget, in his *Physiology*, gives the following as the conditions essential to perfect nutrition: 1. "A right state and composition of the blood. 2. A regular and not far distant supply of such blood. 3. A certain influence of the nervous system. 4. A natural state of the part to be nourished." Fatty degeneration may result from the failure or defect of any one of these conditions.

1. The right state and composition of the blood consists in a certain adaptation between the blood and the tissues, and also each part of every tissue; and upon the maintenance of this perfect adaptation depends the continuance of healthy nutrition. The blood is modified by age—it may be said to grow old. In the aged, we most frequently meet with fatty degeneration of the various tissues. This degeneration, occurring late in life, can hardly be called abnormal more than decay and death in the vegetable world. But degeneration is impatient, and anticipates time. Instances of fatty degeneration have been observed at eight and six years of age; and one case of degeneration of the small cerebral vessels in a child of only nineteen months has been reported. This premature degeneration is often induced and hastened by numerous local and general diseases, in which the blood is impoverished and its composition rendered defective. In anæmia, hemorrhage, Bright's disease, organic disease of the heart, marsh cachexia, and in most chronic diseases, the blood is deficient in the red corpuscles; in Bright's disease, marsh cachexia, cancer, and in the last stage of disease of the heart, the albumen is diminished; and in disease of the heart and chronic scurvy the fibrin is deficient. In lingering and wasting diseases fatty degeneration of a vital organ frequently coexists; a complication full of peril, which should be watched for, and, if possible, detected. During chronic diseases sometimes, upon some slight exertion, a patient turns pale and expires in a moment; the heart is at fault; its fibres are so degenerated they no longer respond to the stimulus of the organic nerves.

A mulatto woman, of about thirty-five years of age, was admitted into the Dexter Asylum in the afternoon; she had been suffering from a severe diarrhœa for some time; during the night she got up to use the close stool, and died instantly. Upon examination, the fibres of the heart were found to have undergone fatty degeneration. It is to be feared that large and frequent losses of blood by the lancet, as well as by disease, have sometimes aided in producing, and often greatly aggravated fatty degeneration when present.

2. A regular and not far distant supply of healthy blood is a condition essential to perfect nutrition. Hence, all general or local obstructions to the circulation must impair nutrition. The supply to any part may be rendered insufficient by obstructions at the inlets and outlets of the heart, acting as partial ligatures on the circulation; by changes in the coats of the arteries, diminishing their caliber; or by clots completely filling them; and by fatty degeneration of the coats of the minute vessels. Whether these local obstructions to the circulation shall result in atrophy, degeneration, or death of the part, depends upon the state of the neighboring vessels, which may be too degenerated themselves to enlarge and supply the necessary blood. Degeneration of the arteries may be so general as to prohibit the application of a ligature to an aneurismal vessel, especially if there is reason to think the heart fatty or feeble. The comparatively frequent fatty degeneration of the heart has been explained by the want of free anastomosis of the coronary arteries.

3. The question of nervous influence upon nutrition is very complex; but to measure the influence of its derangement or withdrawal, in the production of atrophy and fatty degeneration, and to separate it from other causes acting at the same is still more difficult, a still more complex problem. But that the brain does have, not only an indirect but also a direct influence on nutrition, is a matter about which there cannot be much doubt. A depressed state of mind interferes indirectly with nutrition by withholding the body from exercise and relaxation, which are necessary to its perfect health; by weakening the heart's action, so the blood is not circulated with the necessary force and frequency; by lessening the amount of air respired, thus rendering the oxygenation of the blood imperfect; by impairing the digestive process, so an imperfect chyle is supplied to the blood; and, frequently, by preventing or disturbing sleep. That the emotions of the mind directly affect nutrition may be inferred from their known influence upon the different secretions in health. Hence it is highly probable, that deranged nervous influence predisposes to degeneration, and aggravates it when it already exists. Cases of softening of the brain have been recorded, which were mainly attributable to grief and mental anxiety. Dr. Quain has reported several cases of fatty degeneration of the heart, in persons who had suffered much from mental anxiety. Three of these cases resulted in rupture. The expression, "died of a broken heart," in these cases was literally true. Drs. Ormerod, Daniell and others, mention like cases.

Quekett found, from numerous examinations, that simple atrophy, not fatty degeneration, followed the withdrawal of nervous influence in cases of paraplegia from disease or fracture of the spine.

4. The fourth condition necessary to perfect nutrition is a healthy state of the part to be nourished. Each part and tissue has a special life of its own, dependent on the vitality of the whole organism, partaking of its hereditary strength or weakness. Each tissue has its own peculiar and discriminating process of assimilation; it may die prematurely, or remain perfect upon the death of the rest of the body. Assimilation is the formation of new parts, like those already existing; whether the latter be normal or abnormal, the same structure is perpetuated. If a part is healthy, it is likely to remain so; if altered, the alteration is maintained. Thus, the form and size of a cicatrix remain the same for years. But, opposed to this law, there exists in nearly all altered parts a tendency to regain the normal state, and often, in time, this state is recovered; the scar, as it were, wears out.

A simply atrophied tissue can be restored; but whether a tissue, affected with fatty degeneration, can recover its lost structure is yet an unsettled question. There is some reason to believe it can. Mr. Charles Simpson reports a case in which a well-marked arcus senilis disappeared. This is another instance in which the eye affords an opportunity to watch the changes taking place in the body. The fact already mentioned, that the fibres of the uterus, after delivery, undergo fatty degeneration, and new ones are developed when it again resumes its functions, would lead one to think that the process was not one of absolute and irrecoverable destruction. But, even granting that a fibre affected with fatty degeneration is immediately destroyed, degeneration of the remaining may be arrested, and new vigor infused into the organ by enlarging and strengthening the adjoining undestroyed fibres. In the young, the middle-aged, and even in the old, where much uninjured tissue remains, we may reasonably hope to counteract the effects of fatty degeneration by restoring vigor to the surrounding structures. Dr. Quain found very great benefit to follow from proper treatment; greater, of course, in cases of premature decay than in the aged. The length of time the tissues remain perfect in some cases, is not less wonderful than their premature decay in others. Mr. Canton, in his researches with regard to the senile arc, gives the case of a man aged 103. The cornea was free from the arc, and the microscope revealed only a very few fat-granules; in the fibres of the heart, there were only two or three limited spots of fatty degeneration.

It is the vital principle—the life power of Aristotle—the animating principle of Harvey—the organic force of Müller, and the organic agent of Prout, however vague and imperfect our ideas of this principle are; yet it is this power, or combined power of life, that preserves our bodies in integrity, and prevents those changes which we call *death*.

When fatty degeneration occurs as a general disorder, affecting many organs and tissues, it is most frequently met with in those of feeble circulation and imperfect respiration, in the aged, the cachectic, the habitually intemperate, and especially in the scrofulous. So frequently does the tubercular diathesis associate with itself fatty degeneration of certain organs, that Mr. Simon believes that some essential relation exists between them. "Degeneration," says Dr. Prout, "appears to be connected with, or to result from, the gradual decay of the vital processes in general, and particularly of the processes of assimilation. It is, therefore, the natural and universal consequence of age; but it may arise in early life from a variety of causes, among which the most frequent are: 1. An inherited and innate weakness of the vital powers, either as they exist in the system generally, or as they exist in particular organs. 2. An acquired weakness of the vital powers in general, or as regards the vital powers of particular organs, produced by a variety of slowly-acting causes; such as long-continued errors in eating and drinking, long exposure to the influence of unhealthy situations, or of occupations unfavorable to the general health.

When fatty degeneration is limited to one organ, or part, it is usually the effect of some previous disease or accident, which prevents a free supply of blood to the organ or part.

All animal substances, whose elements are held together by the feeblest affinities, soon undergo fatty degeneration. Thus lymph, effused into the lungs, liver, kidney, or testicle, in low chronic forms of inflammation, in gangrene; imperfectly organized lymph upon serous membranes; crude and softened tubercular masses; cancerous growths; fibrinous vegetations on the valves of the heart, and pus, all give the clearest evidence, under the microscope, of being principally made up of fat-granules. The artificial formation of adipocere, from nearly all the soft tissues of the body, most clearly shows that the process of fatty degeneration depends upon the prevailing of the *chemical affinities* over the *vital powers*. Whenever and wherever fatty degeneration occurs, it is the result of mal-nutrition, a process of decay, a degradation of the protein compounds to the lowest in the animal and vegetable kingdoms. It enfeebles the vital and physical properties of all the organs and tissues it affects; by it, the muscles lose their contractility, the heart is enfeebled and dilated, and may be ruptured; it injures the elasticity of the arteries, causes aneurisms and their rupture; it clogs the cells, ducts, and vessels of the secretory organs, and thus interferes with, and sometimes completely prevents the performance of their functions.

There are many more important facts connected with this interesting subject, which have been brought to light by the researches of living pathological microscopists, and published in the various medical journals.

Whatever answers future discoveries may return to such questions as the following: Does the process of fatty degeneration consist in a tendency to deposit oil instead of a healthy tissue, or does the low vitality of the part only admit of the deposit of fat-granules, as the substance possessing the lowest degree of vitality; whether the fat-granules in the degenerated tissue are capable of farther transformation or removal, or whether this must be considered a final stage in the vital processes of the organism?—whatever solution science may hereafter return to such questions as these, the practical importance of this subject to the practising physician must ever be great, not only from the number of organs and tissues it affects, but also from the vast number of pathological conditions it alone explains. He must ever be on the alert to detect it; and, where it exists, it must, to a greater or less extent, modify his practice.

*Treatment.*—From what has already been said upon the causes of fatty degeneration, the course of treatment most likely to stay the progress of the disease, and, if possible, to restore the degenerated tissue, is readily inferred; namely, to sustain the vital powers, and preserve the organic functions in due activity; to improve the condition of the blood, making it rich in highly vitalized fibrin and albumen, by a diet of lean meats, bread, and succulent vegetables, excluding all articles rich in fat, with sparing use of sugar and fermented liquors; to promote free circulation and full respiration, by regular exercise in pure air; to restore healthy action of the skin, by bathing and friction; and to keep up a full and healthy action of the bowels. The tonics, iron, bark, and the mineral acids, especially the nitro-muriatic, have been found to be of decided utility. From the tendency which the fatty matter in degenerations has to assume the solid form, Dr. Williams suggests the use of cod-liver oil as a solvent of the fatty concretions.

